# The fetuses-at-risk approach: Clarification of semantic and conceptual misapprehension

**DOI:** 10.1186/1471-2393-8-11

**Published:** 2008-03-26

**Authors:** K S Joseph

**Affiliations:** 1 Perinatal Epidemiology Research Unit, Departments of Obstetrics & Gynaecology and Pediatrics, Dalhousie University and the IWK Health Centre, Halifax, Nova Scotia, Canada

## Abstract

**Background:**

Although proponents of the fetuses-at-risk approach describe it as a causal model that resolves various conundrums, several areas of semantic and conceptual misapprehension remain. Differences in terminology include use of denominators such as 'ongoing pregnancies' and the need for an ad hoc 'correction factor' in order to calculate gestational age-specific rates. Further, there is conceptual disagreement regarding the proper candidates for neonatal death and related phenomena. Perhaps the most egregious misconception is the belief that rising rates of gestational age-specific perinatal mortality observed under the fetuses-at-risk model automatically imply the need for indiscriminate increases in iatrogenic preterm delivery.

**Discussion:**

The term 'fetuses at risk' addresses the plurality of candidates for stillbirth in a multi-fetal pregnancy, while the use of standard terminology such as 'cumulative incidence' and 'incidence density' harmonizes the language of perinatal epidemiology with that used in the general epidemiologic literature. On the conceptual side, it is necessary to integrate clinical insights regarding latent periods into models of neonatal morbidity and mortality. The contention that the fetuses-at-risk approach implies the need for indiscriminate iatrogenic preterm delivery is a non-sequitur (just as rising age-specific cancer death rates do not imply the need for routine chemotherapy and radiation for all middle aged people). Finally, the traditional and fetuses-at-risk models are better viewed in terms of function as prognostic (non-causal) and causal models, respectively.

**Conclusion:**

A careful examination of terms and concepts helps situate the traditional perinatal and the fetuses-at-risk approaches within the broader context of non-causal and causal models within general epidemiology.

## Background

A recent *Commentary *[1] in the *Journal *addressed methodologic issues related to gestational age and contested several points discussed in my recent paper on obstetric theory [2]. Whereas this debate has served to focus attention on several key issues in perinatology, some areas of semantic confusion and conceptual misapprehension remain.

## Semantic issues

The *Commentary *advocated using 'ongoing pregnancies' as the denominator for calculating the antepartum risk of stillbirth. An alternative term is 'fetuses at risk' for antepartum stillbirth [3-5]. The difference between the two denominators is minor, though the distinction, which recognizes the plurality of candidates for antepartum stillbirth within multi-fetal pregnancies, is necessary from an epidemiologic perspective.

A second semantic issue relates to the details of risk quantification. The number of fetuses at risk for antepartum stillbirth decreases from the beginning to the end of each gestational week and the pattern of this decrease varies before and after 40 weeks gestation. The *Commentary *[1] addressed this by highlighting the need for an ad hoc 'correction factor' [6]. We choose to address the same issue by invoking standard epidemiologic terminology [5], namely, cumulative incidence (the proportion of a fixed population that develops the outcome of interest over a specified time period) and incidence density (the ratio of the number of new cases of the outcome of interest to the person-time at risk) [7].

With these epidemiologic terms defined, it becomes evident that the 'prospective risk of stillbirth' [8] at any gestation is a cumulative incidence, with the duration over which incidence is measured left open ended (similar to the lifetime cumulative incidence of breast cancer). Alternatively, the cumulative incidence of stillbirth at any gestation can be estimated within a specific time window. From an obstetric perspective, a meaningful length for the time interval would encompass the period after a clinical examination during which fetal/maternal status is expected to be stable, with the specific duration dependent on the risk status and gestation of the pregnancy and the clinical assessment in question. With medically indicated early delivery predicated on the short-term risks of serious events, it becomes evident that quantification of an open ended prospective risk of stillbirth is not central to the practice of obstetrics.

## Conceptual issues

The extension of the fetuses-at-risk approach to encompass perinatal death and other phenomena is criticised in the *Commentary *[1] because many such events, exemplified by death in the neonatal period, do not occur among fetuses. Such criticisms can be countered on clinical and epidemiological grounds. The focus in modern obstetrics extends well beyond fetal outcomes and encompasses concerns regarding neonatal death, serious neonatal morbidity and even neuro-developmental disability at 2 years of age [9,10]. This extended focus reflects an appreciation of latent periods (i.e., the time interval between disease occurrence and detection [7]). For instance, it is well recognized that the neurologic injury that characterizes cerebral palsy is typically sustained in utero, despite becoming clinically evident a year or more after birth [11,12]. The extended fetuses-at-risk model therefore proposes that the gestational age-specific rates of outcomes such as cerebral palsy are more appropriately calculated using fetuses as the candidates for cerebral palsy [13]. The same argument applies to neonatal death – the pathological events that result in neonatal death typically occur during the intrauterine period. From an epidemiologic standpoint as well, it is commonplace to estimate cancer and other cause-specific mortality rates by age, and in the perinatal realm, calculations of cause-specific infant mortality (e.g., rate of infant deaths due to congenital anomalies) use all live births in the denominator [14].

Perhaps the single, most serious misunderstanding regarding the fetuses-at-risk approach in the *Commentary *[1] relates to the issue of rising gestational age-specific perinatal mortality rates and the consequent implications for iatrogenic preterm delivery. Although perinatal mortality rates do increase with increasing gestation in fetuses-at-risk models, it is a profound misconception to state that such a pattern automatically implies the need for indiscriminate increases in preterm induction or preterm cesarean delivery. This non-sequitur is analogous to suggesting that those who document the age related rise in cancer mortality advocate routine chemotherapy and radiation for the middle aged.

Selective, carefully-timed, early delivery given fetal compromise (or maternal indication) is the cornerstone of modern obstetrics. Whether early delivery at any gestation can save a compromised fetus depends on the gestational age of the fetus, the degree of compromise and the technologic package available for effecting early delivery and caring for the newborn. Iatrogenic early delivery is carried out at preterm gestation only if the overall risks to the fetus of a continuing pregnancy are judged to exceed those of early delivery and supportive neonatal care. This judgement involves an informal or formal balancing of harms versus benefits [2,5].

The *Commentary *[1] provides a listing of outcomes whose rates are judged to require particular denominators. Our alternative viewpoint posits that the choice of denominator depends on whether one seeks to build a causal or prognostic (non-causal) model [5,13,15]. Fundamental caveats in this context include whether

1) gestational age is to be treated as survival time (causal model) or as just another determinant (prognostic model)

2) the entire biologic continuum from fetus to infant needs to be represented (causal model) or whether a truncated period will suffice (prognostic model)

3) a restrictive approach to variable selection, which avoids variables in the  causal pathway, is deemed appropriate (causal model) or whether a more liberal  approach is considered appropriate (prognostic model).

Thus models that predict neonatal death among live born infants and use determinants such as gestational age can be valid for prognostic purposes and can serve an important social/medical purpose. On the other hand, such models lead to awkward paradoxical phenomena, for example, by consistently showing that preterm infants of smokers have lower mortality rates than preterm infants of non-smokers. These and other conundrums require a causal model for explication [5].

This debate highlights the dichotomy that prevails in the use of fundamental epidemiologic constructs within perinatal epidemiology versus other epidemiologic domains. A careful examination of terms and concepts helps situate the traditional perinatal and the fetuses-at-risk models within the broader context of causal and non-causal models in general epidemiology.

## Competing interests

The author(s) declare that they have no competing interests.

## Pre-publication history

The pre-publication history for this paper can be accessed here:


